# The Impact of the COVID-19 Pandemic on Emergency Department Visits Resulting in Ophthalmology Consultations

**DOI:** 10.7759/cureus.30598

**Published:** 2022-10-23

**Authors:** Ceren Durmaz Engin, Taylan Ozturk, Betul Akbulut Yagci, Oguzhan Ozcelik, Resul Ecer

**Affiliations:** 1 Department of Ophthalmology, Karadeniz Eregli State Hospital, Zonguldak, TUR; 2 Department of Ophthalmology, Dokuz Eylul University, Izmir, TUR; 3 Department of Emergency Medicine, Dokuz Eylul University, Izmir, TUR

**Keywords:** ophthalmology consultation, ophthalmic emergency, lockdown, emergency department, covid-19

## Abstract

Introduction

The aim of this study is to determine the characteristics of eye-related emergency department (ED) visits resulting in ophthalmology consultation during the COVID-19 pandemic and compare them against an equivalent period from the previous year.

Methods

In this study, we reviewed the charts of patients who were admitted to ED with ocular complaints between March 11th, 2020 (the date of the first COVID-positive case in our country) and March 11th, 2021 (Study period 1; SP1) and those who were admitted to ED within the equivalent period of the previous year (Study period 2; SP2). The frequency of eye-related cases, the urgency status of complaints, diagnosis, treatment applied, and hospitalization status of the patients were compared.

Results

The proportion of ophthalmology consultations among all medical departments decreased from 4.52% to 4.04% (p<0.001). There was a 40.5% reduction in eye-related ED admissions during the pandemic, and the top three ocular diagnoses were foreign bodies of the ocular surface (24.3%), corneal abrasion (18.7%), and blow-out fractures (6.2%) during SP1. The proportion of urgent eye-related emergency visits increased during the pandemic year (80.7% of total cases) compared to the year prior to the pandemic (66.0% of total cases) (p<0.001). Although the number of ophthalmology consultations per day decreased during lockdown periods, this decrease was not statistically significant.

Conclusion

During the first year of the COVID-19 pandemic, the number of eye-related ED visits decreased in comparison to the year which preceded the pandemic. However, the proportion of urgent visits increased during the pandemic. Understanding the circumstances under which patients seek eye care in EDs is critical to rendering the optimal level of service of available resources.

## Introduction

The COVID-19 pandemic irreversibly affected many countries across the world, including Turkey. As a result of the precautions taken by the Ministry of Health, most public and private hospitals were declared pandemic hospitals and rapidly restructured their clinical areas and healthcare teams. In order to decrease the acute pressure on the healthcare system, procedures like limiting outpatient clinic visits and delaying elective surgeries were implemented [1]. Nevertheless, emergency departments (ED) operated at full capacity. Due to the limitations of outpatient clinic visits, many patients presented to EDs for their non-urgent complaints. Furthermore, patients with low-grade fever, mild cough, and headaches, which were previously considered mild and recommended to be handled at home, presented to the ED believing they had COVID-19. All of this contributed to the workload of emergency services.

Ophthalmology was among the branches with the highest decrease in the number of outpatients during the pandemic [2]. As in the rest of the world, ophthalmology clinics in our country were recommended to cease providing any treatment other than emergent care. A study conducted in Turkey showed a 56% reduction in the number of patients attending the ophthalmology clinic during the pandemic [3]. During this period, when outpatient clinics were only seeing patients with appointments, patients without appointments were seeking care from the multi-purpose EDs for their eye-related symptoms. Patients were initially examined by an ED physician before being referred to the ophthalmology clinic on the same day. Several studies have found a decrease in visits to ophthalmic emergency departments during the pandemic [4,5]. A few of these studies were conducted in multi-purpose EDs where not only eye-related symptoms but all urgent medical complaints were evaluated, and a few studies quantified the number of visits and the etiologies of eye diseases. Therefore, the results of this study can help to improve triage policies, guide public education, and educate the public on what constitutes urgent ophthalmologic emergencies.

## Materials and methods

This retrospective study was carried out in a tertiary care university hospital in Turkey. Depending on the population of COVID-19-positive patients across the province, restrictions were imposed on the admission of patients to the outpatient clinics on occasion. If the number of COVID-19-positive patients determined on a weekly basis exceeded the threshold set by the Ministry of Health, lockdown measures were implemented in that province, and admission to the hospital was allowed only for emergencies. Otherwise, it was possible to apply to outpatient clinics with non-urgent complaints. During the pandemic, ophthalmic EDs for patients with emergent ocular symptoms were only available in a few eye-specific hospitals in Turkey, therefore, in many tertiary hospitals, ocular emergencies were accepted by the hospital's multi-purpose ED and referred to the on-call ophthalmologist.

This study adhered to the ethical standards in the Declaration of Helsinki. Ethical approval was received from the local ethics committee of Dokuz Eylul University (approval number: 2021/10-49).

Study population

A retrospective chart review of ED referrals between March 11, 2020, and March 10, 2021 (one year from the onset of the first COVID-positive case in Turkey) and from March 11, 2019, to March 10, 2020 (the previous year's equivalent time interval) was conducted and referred to as “Study Period 1 (SP1)" and "Study Period 2 (SP2)", respectively. Data included patient age and gender, time of admission, ophthalmic diagnosis, administered treatment, and hospitalization status. Based on the ophthalmic diagnosis, consultations were categorized as likely emergent, possibly non-emergent, and unidentified [6]. Patients with missing data and those who left the hospital without being examined by an ophthalmologist were excluded. Furthermore, in order to understand the impact of the lockdown, the consultations were divided into three blocks: 11 March to 3 June 2020 (1st lockdown), 4 June to 17 November 2020 (No lockdown), and 18 November 2020 to 10 March 2021 (2nd lockdown) according to restrictions for "walk-in" patients secondary to the national lockdown measures.

Statistical analysis

Statistical analysis was performed with SPSS Statistics v. 23 (IBM Corp., Armonk, NY). Numerical variables were represented as mean ± standard deviation or median (interquartile range), and categorical variables as counts and percentages. Independent sample t-test was used for variables with normal distribution and Mann-Whitney U test was used for non-normally distributed variables. The chi-square test assessed categorical variable relationships. Multiple linear regression to determine factors affecting emergent visits was performed. A comparison of the two rates between study periods was performed with MedCalc Statistical Software version 19.2.6 (MedCalc Software bv, Ostend, Belgium). The statistical significance level was set as p < 0.05.

## Results

Distribution of consultations according to medical departments

**Figure 1:**
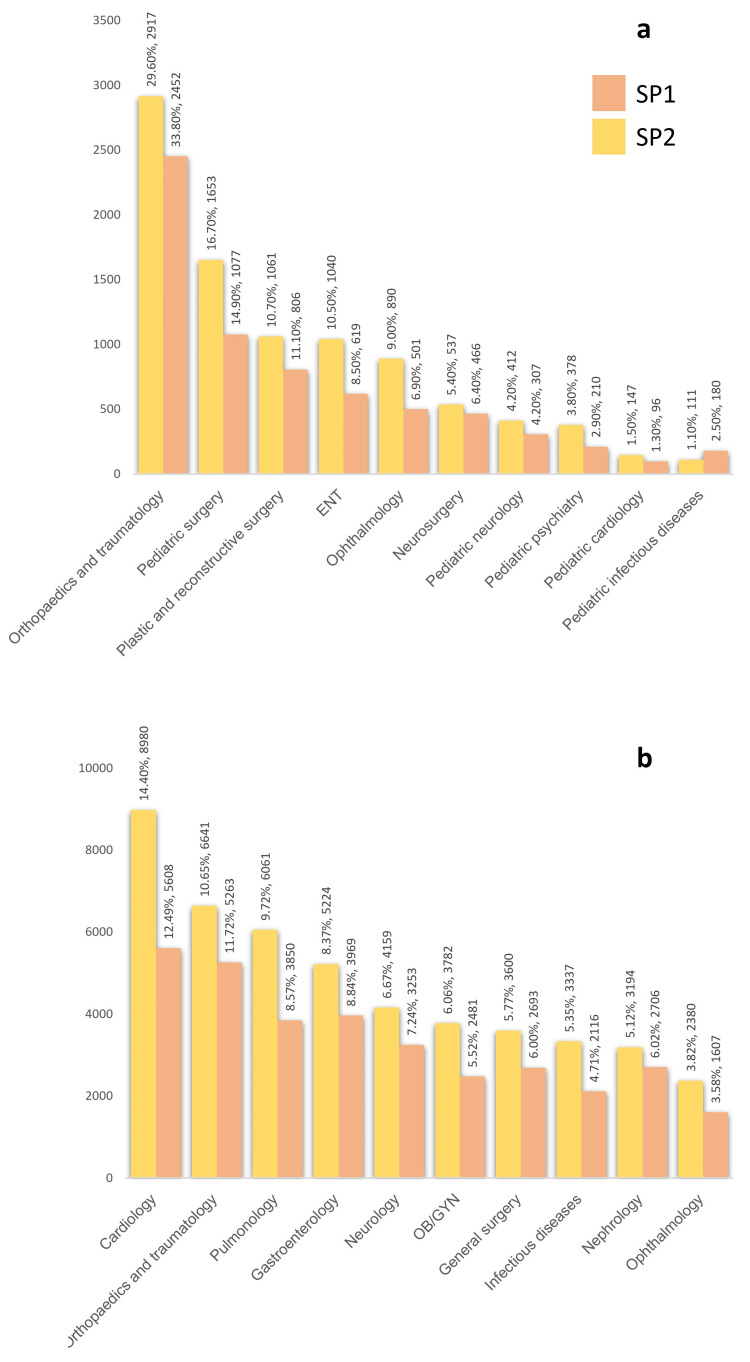
Changes in the number and proportion of consultations for different medical departments in children (a) and adults (b) between Study Period 1 (SP1) and Study Period 2 (SP2).

The number of consultations from ED in SP1 was 44917 for adults and 7251 for children, compared to 62378 and 9870 in SP2, respectively. The proportion of ophthalmology consultations among all medical departments decreased from 4.52% to 4.04% (odds ratio [OR], 0.89; 95% CI, 0.84-0.94) (p<0.001). The distribution of consultations by medical departments is shown in Figure 1.

The number of ophthalmology consultations was 2108 in SP1 (1607 adults and 501 children), while this number was 3270 (2380 adults and 890 children) in SP2. When these consultations were analyzed by retrospective chart review, it was seen that a total of 361 (17.1%) patients in SP1 and 337 (10.2%) patients in SP2 either did not show up or had a lack of data in their charts. After excluding those, the total number of consultations included in the study was 1747, including 383 children and 1364 adults in SP1; and 2933, of which 724 were children and 2209 were adults in SP2. There was a 40.5% reduction in eye-related referrals from ED between SP1 and SP2 for all study populations.

Patient demographics

The mean age of the pediatric and adult patients in SP1 was 7.85 years (0 - 18) and 44.3 years (19 - 95), respectively, while it was 8.01 years (0 - 18) in pediatric and 45.17 years (19 - 95) in the adult population in SP2. The majority of patients were between the ages of 20 and 39 and constituted 31.4% and 33.5% of the patients in SP1 and SP2, respectively. Male patients made up a higher proportion of referrals in SP2 (58.2% among pediatric and 66.6% among adult population), and a significant increase was observed in the proportion of male patients in SP1 for adults (74.4% of all population, p<0.001) but not for children (63.2% of all population, p=0.160).

Diagnosis and urgency status

The proportion of likely emergent referrals increased to 72.6% (n=278) in children and 83.0% (n=1132) in adults in SP1 (p<0.001 for both). The time of day at which patients were admitted did not change significantly between SP1 and SP2 (p=0.085 for children and p=0.250 for adults). Table 1 summarizes the demographic characteristics of study patients and the patterns of consultations.

**Table 1:** Demographic characteristics of consulted pediatric and adult patients in both study periods SP1: Study Period 1; SP2: Study Period 2 * Invasive procedures that do not require operating room conditions (removal of corneal and conjunctival foreign bodies, laser for retinal tear etc.)

	Pediatric Patients			Adult Patients		
	SP1	SP2	p	SP1	SP2	p
Gender (n,%)						
Female	141 (36.8%)	298 (41.2%)	0.160	349 (25.6%)	737 (33.4%)	<0.005
Male	242 (63.2%)	426 (58.8%)		1015 (74.4%)	1472 (66.6%)	
Time of Admission (n,%)						
12 PM to 8 AM	50 (13.1%)	64 (8.8%)	0.085	233 (17.1%)	334 (15.1%)	0.250
8 AM to 5 PM	132 (34.5%)	254 (35.1%)		525 (38.5%)	891 (40.3%)	
5 PM to 12 PM	201 (52.5%)	406 (56.1%)		606 (44.4%)	984 (44.5%)	
Diagnostic Category (n,%)						
Not Likely Emergent	90 (23.5%)	300 (41.4%)	<0.005	189 (13.9%)	578 (26.2%)	<0.005
Likely Emergent	278 (72.6%)	388 (53.6%)		1132 (83.0%)	1549 (70.1%)	
Not Determined	15 (3.9%)	36 (5.0%)		43 (3.2%)	82 (3.7%)	
Treatment (n,%)						
No treatment	49 (12.8%)	113 (15.6%)	0.038	161 (11.8%)	344 (15.6%)	<0.005
Medical	282 (73.6%)	528 (74.3%)		683 (50.1%)	1239 (56.1%)	
Invasive (outpatient)*	39 (10.2%)	42 (5.8%)		421 (30.9%)	515 (23.3%)	
Surgery	13 (3.4%)	31 (4.3%)		99 (7.3%)	111 (5.0%)	
Hospitalization status (n,%)						
No	358 (93.5%)	658 (90.9%)	0.136	1246 (91.3%)	2063 (93.4%)	0.023
Yes	25 (6.5%)	66 (9.1%)		118 (8.7%)	146 (6.6%)	

**Figure 2:**
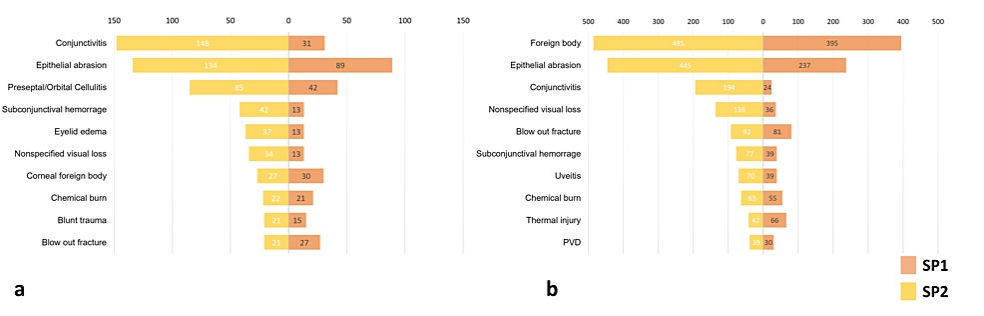
The most common 10 diagnostic groups in children (a) and adults (b) as a result of ophthalmology consultations in Study Period 1 (SP1) and Study Period 2 (SP2).

In the pediatric population, the most common diagnosis in SP1 was "epithelial abrasion" (n=89, 23.2%), while it was "conjunctivitis" (n=148, 20.4%) in SP2. "Foreign body of ocular surface" was the most common diagnosis for adults in both study periods (n=395, 29.0% in SP1 and n=485, 22% in SP2). Figure 2 shows the most common diagnoses established in pediatric and adult patients.

The prevalence ratio (PR) was calculated for each diagnostic group as the proportion of consultations in SP1 divided by the equivalent proportion in SP2. Examining the PR of diagnostic groups across the years, we observed that thermal injuries (including flash burns from welding arch), optic neuropathy, and blow-out fractures were more frequent during SP1, whereas other likely non-emergent diseases such as conjunctivitis and eyelid disorders were less prevalent. Although the PR of diagnostic groups such as acute endophthalmitis (PR=2.33), open globe injury (PR=1.43), and retinal detachment (PR=1.40) was above 1, they did not reach statistical significance due to the low number of cases. The PRs of diagnostic groups in SP1 are given in Tables 2-3.

**Table 2:** Diagnostic groups that are more prevalent in Study Period 1 (SP1) compared to the previous year (PR > 1.00) CI: confidence interval; LE: likely emergent; PNE: possibly non-emergent; PR: prevalence ratio *Diagnostic categories reaching statistical significance

Emergency Status	Diagnosis	PR	95% CI	p
LE	Penetration of eyeball with foreign body	2.79	0.54 to 18.01	0.17
LE	Thermal injury*	2.78	1.91 to 4.09	< 0.0001
LE	Acute endophthalmitis	2.39	0.82 to 7.42	0.07
LE	Transient arterial occlusion	2.09	0.74 to 6.11	0.12
LE	Papilledema	1.83	0.73 to 4.58	0.15
LE	Optic neuropathy*	1.81	1.01 to 3.25	0.03
LE	Orbital blow out fracture*	1.60	1.22 to 2.10	0.0005
LE	Chemical burn*	1.50	1.08 to 2.07	0.01
LE	Retinal detachment	1.48	0.87 to 2.49	0.12
LE	Retinal tear	1.43	0.39 to 5.00	0.51
LE	Foreign body of ocular surface*	1.39	1.22 to 1.58	< 0.0001
LE	Herpetic keratitis	1.28	0.57 to 2.80	0.49
LE	Laceration of the skin of the eyelid and periocular area	1.23	0.75 to 2.01	0.36
PNE	Blepharitis	1.22	0.69 to 2.12	0.45
LE	Open globe injury (without foreign body)	1.19	0.72 to 1.96	0.44

**Table 3:** Diagnostic groups that were less prevalent in Study Period 1 (SP1) compared to the previous year (PR < 1.00) CI, confidence interval; LE, Likely emergent; PNE, possibly non-emergent; PR, prevalance ratio; U/I, unidentified *Diagnostic categories reaching statistical significance

Emergency Status	Diagnosis	PR	95% CI	p
NLE	Conjunctivitis*	0.27	0.19 to 0.35	< 0.001
LE	Periocular area abrasion*	0.29	0.07 to 0.85	0.01
U/I	Episcleritis	0.35	0.06 to 1.28	0.09
NLE	Dry eye disease	0.39	0.09 to 1.21	0.08
LE	Acute dacryocystitis	0.41	0.04 to 2.10	0.27
NLE	Unspecified visual loss*	0.48	0.34 to 0.66	< 0.001
NLE	Edema of eyelid*	0.57	0.30 to 1.02	0.04
LE	Vitreous hemorrhage	0.64	0.30 to 1.30	0.20
U/I	Headache	0.64	0.30 to 1.30	0.20
LE	Retinal vascular occlusion	0.67	0.15 to 2.32	0.52
NLE	Subconjunctival hemorrhage*	0.73	0.51 to 1.02	0.05
LE	Preseptal cellulitis	0.77	0.55 to 1.06	0.10
U/I	Surgery related problems	0.83	0.43 to 1.57	0.57
NLE	Blunt trauma to eye w/o perforation	0.93	0.59 to 1.46	0.78
EM	Epithelial abrasion of ocular surface	0.94	0.82 to 1.08	0.41

The results of a multivariate logistic regression model showed that emergent consultations in SP1 were most likely among males (odds ratio [OR], 2.55; 95% CI, 1.99-3.27), patients younger than 65 years of age (OR, 1.74; 95% CI, 1.22-2.49), and patients who presented to ER outside of working hours (OR, 1.38; 95%CI, 1.08-1.77).

Treatment and hospitalization

In both study periods, the most frequent treatment modality was “medical treatment” in pediatric (n=282, 73.6% for SP1; n=538, 74.3% for SP2) and adult (n=683, 50.1% for SP1; n=1239, 56.1% for SP2) patients. Removal of foreign body was the most commonly performed invasive procedure (92.2% and 91.6% of all invasive procedures in SP1 and SP2, respectively). For the entire study population, the most common diagnoses that resulted in surgical treatment in SP1 were open globe injuries (28.3% of all surgeries), retinal detachment (25.2% of all surgeries), and skin laceration of the eyelid and periocular area (9.4% of all surgeries).

Contrary to decreased pediatric hospitalization rates for ocular problems (6.5% for SP1 and 9.1% for SP2; p=0.136), increased adult hospitalization rates were found during the pandemic (6.6% in SP2 and 8.7% in SP1, p=0.23). Leading diagnoses that resulted in hospital admissions included open globe injuries (20.3% of all admissions), retinal detachment (18.0% of all admissions), and orbital cellulitis (13.8% of all admissions) for the entire study population.

Effect of lockdown

**Figure 3:**
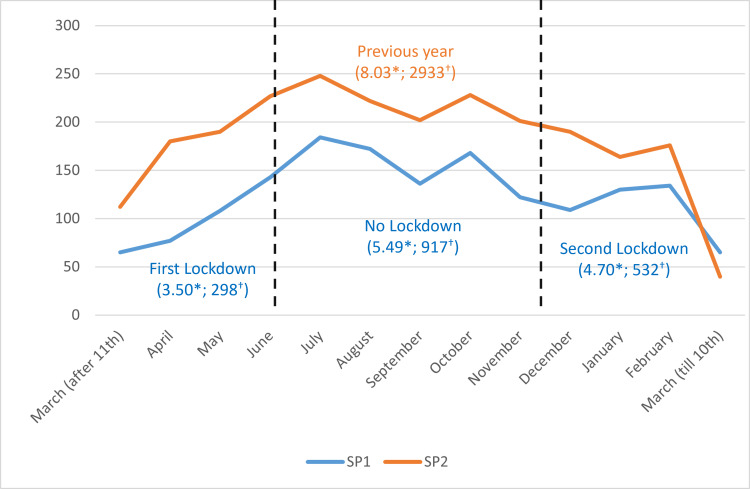
The effect of lockdown on the change in total (†) and daily consultation (*) numbers during Study Period 1 (SP1) compared to the previous year (Study Period 2; SP2)

Although the number of consulted patients decreased during lockdowns, no statistically significant decrease was found in the average number of daily consultations in the first lockdown (=1.87; p=0.170) and second lockdown (=0.42; p=0.510) compared to the “No lockdown” period. Figure 3 shows the change in the total and daily consultation numbers in each period. There was no difference in terms of the gender of the patients (p=0.783), urgency status (p=0.956), admission time (p=0.154), and hospitalization rates (p=0.898) between the three periods examined during the pandemic.

## Discussion

Identifying the characteristics of ophthalmology consultations at a tertiary care center is valuable for preparing for emergencies and establishing appropriate triage protocols during the pandemic. In this way, the emergency services, whose burden has increased considerably due to Covid, can be used more efficiently. In our study, while ophthalmology maintained its place among the referrals to all medical departments, its rate decreased from 9.0% to 6.0% in children and from 3.83% to 3.58% in adults. Another study from Turkey showed a decrease of around 50% in total ED visits and 30% in ED consultations, and the proportion of ophthalmology decreased from 2.89% to 1.57 in adults among all departments [7].

We found an overall 40.5% decrease in ED referrals to ophthalmology clinics during the COVID-19 period. This is probably due to social guidelines that advised people to stay home and the fear of exposure to Covid-19 in the hospital, resulting in a higher threshold for visiting the emergency room [8]. From March 15 to April 25 in 2020, the number of people using Moorfields Eye Hospital's emergency services dropped by 60% compared to the previous year [9]. Another study from Spain found a 65% drop in ophthalmic ED visits from March to June, 2020 compared to 2017 [10]. The lower rate in our study may be due to the fact that ours covers the entire first year of the pandemic, not just the initial months when people are most hesitant to seek treatment.

A study from Australia reported that eye-related emergency service visits, which decreased during the lockdown period at the beginning of the pandemic, returned to the same level in 2019 in the post-lockdown period [11]. Another study from the United States showed that outpatient, ED, and hospital admissions dropped by up to 60% from February to April 2020, then rebounded after June [12]. Although not statistically significant, we also observed a similar pattern following the lockdown period. Anxiety about getting to the hospital when the disease is more severe, as well as transportation issues caused by restrictions imposed during lockdown periods, can all contribute to this.

We expected fewer elderly admissions due to the high risk of COVID-19 complications in this population. However, the percentage of patients aged 65 years and older was 11.4% (n=334) among all patients in 2019, compared to 10% (n=174) during the pandemic (p=0.129). The rate of pediatric patients decreased from 24.6% to 21.9%, as we expected, secondary to the reduction of transmitted diseases such as conjunctivitis due to the closure of schools (p=0.051).

Emergent diagnoses increased in both children and adults during the pandemic. We found a significant increase in the rate of thermal damage to the ocular surface, especially due to welding arch injuries in adults. In Turkey, while civil servants or office employees were permitted to work in rotation or from home; factory or construction workers continued to work during the pandemic. This may explain the increased rate of both thermal damage and orbital fractures. Similar to previous studies, we found a significant decrease in conjunctivitis cases in SP1 [11,13]. Measures to stop the spread of Covid-19 such as awareness of hand hygiene practices, "shielding", social distancing measures, and school closures could have a role in reducing the spread of conjunctivitis [14]. In addition, increased disinfectant and cleaning agents used during the pandemic may be associated with an increased rate of chemical damage to the ocular surface, which was most commonly seen in children aged 0-10 (22.3%) in our study. We also examined a lot of children who had splashed disinfectant in their eyes. There are other studies reporting increased disinfectant-induced ocular damage in children, and although no long-term sequelae were observed in any case, including our study, disinfectant use should be supervised by an adult in this age group, or alternative methods of hand hygiene may be preferred [15].

Many ophthalmology clinics have reported reductions of up to 90% in the number of surgical procedures [16,17]. The decrease in the mentioned studies was due to the decrease in elective surgeries. On the other hand, we observed that the proportion of interventional procedures increased during the pandemic period in our study. This, we believe, is because our study population is composed entirely of patients referred from ED, excluding outpatient clinics.

This retrospective study has limitations. Data were collected from electronic medical records; therefore, it is dependent on accurate record keeping. Our data may not be generalized to worldwide multi-purpose EDs. Many ocular manifestations caused by Covid have been described. As we did not test our patients for Covid-19, we cannot comment if the ocular findings could have been related to a Covid-19 infection, however, this situation is outside the scope of our study.

## Conclusions

Our study can serve as a framework for ED and ophthalmology clinics to prepare for possible future pandemics. Understanding the distribution of patients admitted to the ED for ocular problems is important to ensure the optimum use of our available resources. Another area of ​​improvement could be to increase access to expedited diagnosis and treatment for patients' complaints that they consider urgent. For instance, rapid-access ophthalmic emergency clinics would allow patients to avoid the emergency room, thereby minimizing their risk of exposure and decreasing the volume of ED visits. Educating the general public and primary healthcare clinicians about diseases and high-risk situations in eye care will be critical in terms of workload, the danger of contamination of healthcare workers, and the use of healthcare providers' resources.
